# Extracorporeal cardiopulmonary resuscitation without target temperature management for out-of-hospital cardiac arrest patients prolongs the therapeutic time window: a retrospective analysis of a nationwide multicentre observational study in Japan

**DOI:** 10.1186/s40560-020-00478-9

**Published:** 2020-08-03

**Authors:** Maki Kitada, Tadashi Kaneko, Shu Yamada, Masahiro Harada, Takeshi Takahashi

**Affiliations:** 1 Emergency and Critical Care Center, Kumamoto Medical Center, Kumamoto, Japan; 2 Emergency and Critical Care Center, Mie University Hospital, 2-174 Edobashi, Tsu, 514-8507 Japan

**Keywords:** No-flow time, Low-flow time, Neurological outcome, Cerebral performance category

## Abstract

**Background:**

Extracorporeal cardiopulmonary resuscitation (ECPR) with extracorporeal membrane oxygenation (ECMO) is a promising therapy for out-of-hospital cardiac arrest (OHCA) compared with conventional cardiopulmonary resuscitation (CCPR). The no and low-flow time (NLT), the interval from collapse to reperfusion to starting ECMO or to the return of spontaneous circulation (ROSC) in CCPR, is associated with the neurological outcome of OHCA. Because the effects of target temperature management (TTM) on the outcomes of ECPR are unclear, we compared the neurological outcomes of OHCA between ECPR and CCPR without TTM.

**Methods:**

We performed retrospective subanalyses of the Japanese Association for Acute Medicine OHCA registry. Witnessed cases of adult cardiogenic OHCA without TTM were selected. We performed univariate, multivariable and propensity score analyses to compare the neurological outcomes after ECPR or CCPR in all eligible patients and in patients with NLT of > 30 min or > 45 min.

**Results:**

We analysed 2585 cases. Propensity score analysis showed negative result in all patients (odds ratio 0.328 [95% confidence interval 0.141–0.761], *P* = 0.010). However, significant associated with better neurological outcome was shown in patients with NLT of > 30 min or > 45 min (odds ratio 2.977 [95% confidence interval 1.056–8.388], *P* = 0.039, odds ratio 5.099 [95% confidence interval 1.259–20.657], *P* = 0.023, respectively).

**Conclusion:**

This study revealed significant differences in the neurological outcomes between ECPR and CCPR without TTM, in patients with NLT of > 30 min.

## Background

Extracorporeal membrane oxygenation (ECMO) is frequently used as rescue therapy for cardiac arrest, called extracorporeal cardiopulmonary resuscitation (ECPR). ECPR is a promising strategy for managing out-of-hospital cardiac arrest (OHCA) [1–4] and is more effective than conventional cardiopulmonary resuscitation (CCPR).

Recent studies have examined which ECPR factors are related to the patient’s outcome [5–10]. In particular, age, witnessed cardiac arrest, bystander cardiopulmonary resuscitation (BCPR) and initial shockable rhythm (ventricular fibrillation [VF] or ventricular tachycardia [VT]) were strongly associated with improvements in mortality and neurological outcomes, and are therefore included in the indications for ECPR in most departments. Currently, ECPR should be initiated < 60 min after the patient collapses [4, 5].

The interval from collapse to starting the ECMO pump, the no-low time and low-flow time, is an important factor associated with the neurological outcome [11, 12]. In CCPR, the no and low-flow time (NLT) is defined as the interval from collapse to return of spontaneous circulation (ROSC) in the present study (no-flow time: collapse to start cardiopulmonary resuscitation, low-flow time: start cardiopulmonary resuscitation to ROSC).

Realistically, the NLT tends to be longer with ECPR than with CCPR because of the complexity of ECPR, especially the need to insert catheters. However, ECPR is associated with improved outcomes; therefore, it is possible that ECPR could prolong the target NLT. Accordingly, when comparing the neurological outcomes of ECPR and CCPR, it is important to adjust for the NLT.

Combining ECPR with target temperature management (TTM) may also improve the neurological outcomes of OHCA. However, there is not enough evidence to perform TTM in all patients who undergo ECPR [13, 14]. Accordingly, studies investigating the effect of ECPR could exclude any cases of ECPR with TTM to avoid the potential confounding effects of TTM on the outcomes of ECPR.

In Japan, a nationwide observational registry of OHCA was established by the Japanese Association for Acute Medicine (JAAM-OHCA registry), started from June 2014, that includes about 3.7% of ECPR cases [15]. We performed a nationwide, observational study of OHCA cases registered between 2014 and 2018 to retrospectively examine the effectiveness of ECPR.

To confirm the effects of ECPR on neurological outcomes, we excluded patients who underwent TTM to eliminate possible bias by TTM. We also excluded unwitnessed cases because the NLT could not be accurately determined. Multivariable analyses and propensity score analyses were performed using these explanatory factors to assess whether ECPR is associated with significant improvements in neurological outcomes.

## Methods

### Study design

In this study, we used the JAAM-OHCA registry, a prospective registry of OHCA patients at 288 Japanese critical care centres. The registry was approved by the ethics committees at Kyoto University, the participating institutions and each hospital. We retrieved cases registered between June 2014 and December 2017, for retrospective analyses.

### Patients

Between June 2014 and December 2017, there were 34,754 cases of OHCA registered in the JAAM-OHCA registry. We retrieved data for patients who satisfied the following criteria: (1) witnessed collapse with OHCA; (2) age > 18 years; (3) cardiogenic cause of OHCA; (4) ECMO started or ROSC and hospitalisation and (5) no TTM.

### Study outcomes and statistical analysis

The neurological outcomes were assessed in all patients using the Glasgow–Pittsburgh cerebral performance category (CPC), which includes five categories: CPC 1 (good recovery), CPC 2 (moderate disability), CPC 3 (severe disability), CPC 4 (vegetative state) and CPC 5 (death) [16]. We defined a favourable neurological outcome as a CPC of 1–2 at 1 month after collapse.

Amongst 2585 eligible patients, ECPR was performed in 307 and CCPR was performed in 2278. The patients’ age, sex, BCPR, shockable rhythm (VF/VT) and NLT were retrieved from the database as potential confounding factors for the outcome of ECPR.

The patients were divided into those with favourable (CPC 1–2) or unfavourable (CPC 3–5) outcomes. Both of these groups were compared using univariate and multivariable analyses. Univariate analyses were performed with the Mann−Whitney *U* test or Fisher’s exact test, as appropriate. Multivariable analyses were performed using logistic regression analysis, in which the dependent variable was a favourable neurological outcome (CPC 1–2) and the independent variables were age, sex (male), BCPR, shockable rhythm (VF/VT) as the initial rhythm, NLT and ECPR. NLT was defined as the interval from witnessed OHCA to reperfusion (start of ECMO in ECPR or the ROSC in CCPR). These variables were analysed in all eligible patients.

Propensity score analysis was performed by taking into account age, sex (male), BCPR, shockable rhythm (VF/VT) as the initial rhythm and NLT using the inverse probability of the treatment-weighting (IPTW) method, to compare favourable outcome (CPC 1–2) between ECPR and CCPR groups in all, NLT > 30 min, and > 45 min of eligible patients. Furthermore, the patients with NLT ≤ 30 min were also analysed. Propensity score analysis with IPTW methods was calculated by cases in all, NLT > 30 min and > 45 min of eligible patients.

Multivariable analyses were also performed after dividing the patients according to the NLT (> 30 min and > 45 min) for ECPR and CCPR cases separately (the target of initiation ECPR is thought as within 60 min, then 30 and 45 min were treated as cutoff values). Again, we performed logistic regression analysis in which the dependent variable was a favourable neurological outcome (CPC 1–2) and the independent variables were age, sex (male), BCPR, shockable rhythm (VF/VT) as the initial rhythm and NLT.

In all analyses, a *P* value of < 0.05 was considered statistically significant. All statistical analyses, except for propensity score analysis, were performed with SPSS version 25.0 (IBM, Armonk, NY, USA). Propensity score analysis with IPTW method was performed with the R software version 4.0.1 (GNU general public licence).

## Results

**Fig. 1:**
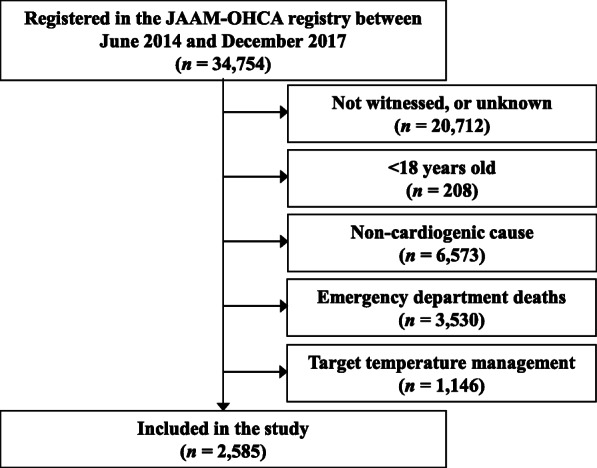
Patient disposition. A total of 2585 patients were considered eligible after applying the inclusion criteria: witnessed out-of-hospital cardiac arrest, age > 18 years, cardiogenic cause, survival in the emergency department, and no target temperature management. JAAM-OHCA, Japanese Association of Acute Medicine, Out-of-Hospital Cardiac Arrest

The registry comprised 34,754 patients, of which 2585 met the inclusion criteria (i.e. witnessed OHCA, age > 18 years, cardiogenic cause, hospitalisation and no TTM; Fig. 1). On the step of eliminate TTM received cases, the inclusion rates of each group were 53% (307/575) in ECPR cases and 72% (2278/3156) in CCPR cases.

Table 1 shows the characteristics of the ECPR and CCPR cases. Overall, 307 patients received ECPR and 2278 patients received CCPR. Multivariable analysis revealed significant differences between the two groups in terms of age, sex (male), shockable rhythm, NLT and favourable outcome.

**Table 1:** Univariate and multivariable comparisons of the ECPR and CCPR groups

Variables	ECPR (*n* = 307)	CCPR (*n* = 2278)	Univariate *P* value	Multivariable *P* value	OR (95% CI)
Age (years)	61 (48–70)	77 (66–85)	< 0.001	< 0.001	0.939 (0.929–0.949)
Male (%)	257 (84%)	1457 (64%)	< 0.001	0.011	1.683 (1.129–2.508)
BCPR (%)	156 (51%)	991 (44%)	0.017	0.723	1.059 (0.772–1.454)
SR (%)	216 (70%)	463 (20%)	< 0.001	< 0.001	9.060 (6.501–12.627)
NLT (min)^a^	60 (50–71)	36 (21–50)	< 0.001	< 0.001	1.056 (1.047–1.065)
CPC 1–2 (%)	22 (7%)	390 (17%)	< 0.001	0.004	0.408 (0.223–0.747)

Values are median (interquartile range) or *n* (%) of cases

*ECPR* extracorporeal cardiopulmonary resuscitation, *CCPR* conventional cardiopulmonary resuscitation, *OR* odds ratio, *CI* confidence interval, *BCPR* bystander cardiopulmonary resuscitation, *SR* shockable rhythm, *NLT* no and low-flow time, *CPC* cerebral performance category

^a^Defined as the interval from witnessed OHCA to start of reperfusion (start of extracorporeal membrane oxygenation for ECPR or return of spontaneous circulation for CCPR)

Table 2 compares the patients divided according to whether their neurological outcomes were favourable (CPC 1–2) or unfavourable (CPC 3–5) amongst all eligible patients. The multivariable analysis revealed significant differences between the two groups in terms of age, BCPR, shockable rhythm, NLT and ECPR. Although the percentage of patients who underwent ECPR was lower in the favourable outcome group, the multivariable analysis showed a positive effect of ECPR on neurological outcomes (odds ratio [OR] 3.782 [95% confidence interval [CI] 1.852–7.723], *P* < 0.001).

**Table 2:** Univariate and multivariable comparisons of cases with favourable and unfavourable neurological outcomes

Variables	Favourable outcomes (CPC 1–2; *n* = 412)	Unfavourable outcomes (CPC 3–5; *n* = 2173)	Univariate *P* value	Multivariable *P* value	OR (95% CI)
Age (years)	64 (52–74)	77 (66–85)	< 0.001	< 0.001	0.947 (0.935–0.958)
Male (%)	312 (76%)	1402 (65%)	< 0.001	0.689	1.076 (0.751–1.543)
BCPR (%)	241 (58%)	906 (42%)	< 0.001	< 0.001	3.109 (2.221–4.352)
SR (%)	203 (49%)	476 (22%)	< 0.001	< 0.001	4.572 (3.184–6.564)
NLT (min)^a^	11 (5–18)	43 (30–56)	< 0.001	< 0.001	0.860 (0.847–0.874)
ECPR (%)	22 (5%)	285 (13%)	< 0.001	< 0.001	3.782 (1.852–7.723)

Values are median (interquartile range) or n (%) of cases

*CPC* cerebral performance category, *OR* odds ratio, *CI* confidence interval, *BCPR* bystander cardiopulmonary resuscitation, *SR* shockable rhythm, *NLT* no and low-flow time, *ECPR* extracorporeal cardiopulmonary resuscitation

^a^Defined as the interval from witnessed OHCA to start of reperfusion (start of extracorporeal membrane oxygenation for ECPR or return of spontaneous circulation for CCPR)

Table 3 shows the comparison of favourable outcome (CPC 1–2) between ECPR and CCPR groups by propensity score analysis with IPTW method, in all, NLT > 30 min and > 45 min cases. In all patients, ECPR shows a negative result for favourable outcome (CPC 1–2) (OR 0.328 CI [0.141–0.761], *P* = 0.010). In patients with NLT of > 30 min and 45 min, show positive results for favourable outcome (CPC 1–2) (OR 2.977 CI [1.056–8.388], *P* = 0.039 and OR 5.099 CI [1.259–20.657], *P* = 0.010, respectively). In patients with NLT of ≤ 30 min, show negative result for favourable outcome (CPC 1–2) (OR 0.174 CI [0.033–0.917], *P* = 0.040).

**Table 3:** Comparison of the proportion of patients with a favourable neurological outcome (CPC 1–2) between the ECPR and CCPR groups using propensity score analysis with the inverse probability of the treatment-weighting method

Variables	Treatment	*n*	CPC 1–2	OR (95% CI)	*P* value
All patients	ECPR	307	22 (7%)	0.328 (0.141–0.761)	0.010
	CCPR	2278	390 (17%)		
NLT (min)^a^					
> 30 min	ECPR	296	17 (6%)	2.977 (1.056–8.388)	0.039
	CCPR	1362	20 (2%)		
> 45 min	ECPR	248	10 (4%)	5.099 (1.259–20.657)	0.023
	CCPR	719	6 (1%)		
NLT					
< 30 min	ECPR	9	4 (44%)	0.174 (0.033–0.917)	0.040
	CCPR	914	370 (40%)		

The propensity score analysis incorporated the following variables: age, sex (male), bystander cardiopulmonary resuscitation, shockable rhythm and low flow time

*CPC* cerebral performance category, *ECPR* extracorporeal cardiopulmonary resuscitation, *CCPR* conventional cardiopulmonary resuscitation, *OR* odds ratio, *CI* confidence interval, *NLT* no and low-flow time

^a^Defined as the interval from witnessed OHCA to start of reperfusion (start of extracorporeal membrane oxygenation for ECPR or return of spontaneous circulation for CCPR)

Tables 4 and 5 show the results of multivariable analyses according to NLT for all patients, patients with an NLT of > 30 min or patients with an NLT of > 45 min for cases who underwent ECPR (Table 3) or CCPR (Table 4), separately. Amongst ECPR cases, the outcome was classified as favourable (CPC 1–2) in 7% of all cases, 6% of cases with NLT > 30 min, 4% of cases with NLT > 45 min. NLT was significantly and independently associated with CPC 1–2 in each subgroup, with an OR of 0.890, 0.898 and 0.855 in each subgroup. Amongst CCPR cases, CPC 1–2 was achieved in 17% of all cases, 2% of cases with NLT > 30 min and 1% in cases with NLT of > 45 min. NLT was significantly associated with CPC 1–2 in the multivariable analysis in all cases of CCPR and in CCPR cases with an NLT of > 30 min, with OR of 0.858 and 0.951, respectively.

**Table 4:** Multivariable analysis of favourable neurological outcome in all ECPR cases and in patients divided by NLT

Variables	All cases (*n* = 307)	*P* value	OR (95% CI)	NLT > 30 min (*n* = 296)	*P* value	OR (95% CI)	NLT > 45 min (*n* = 248)	*P* value	OR (95% CI)
Age (years)	61 (48–70)	0.121	0.973 (0.941–1.007)	61 (48–69)	0.220	0.978 (0.943–1.014)	60 (47–69)	0.800	0.994 (0.945–1.045)
Male (%)	257 (84%)	0.254	0.497 (0.150–1.652)	247 (83%)	0.246	0.491 (0.147–1.635)	208 (84%)	0.018	0.188 (0.047–0.755)
BCPR (%)	156 (51%)	0.927	1.053 (0.351–3.153)	156 (53%)	0.779	1.173 (0.386–3.561)	140 (57%)	0.261	2.383 (0.524–10.849)
SR (%)	216 (70%)	0.017	7.253 (1.428–36.843)	211 (71%)	0.083	4.042 (0.833–19.605)	181 (73%)	0.640	1.492 (0.278–7.999)
NLT (min)^a^	60 (50–71)	<0.001	0.890 (0.848–0.935)	60 (50–72)	< 0.001	0.898 (0.852–0.948)	63 (55–74)	0.010	0.855 (0.760–0.963)
CPC 1–2	22 (7%)	–	–	17 (6%)	–	–	10 (4%)	–	–

Values are median (interquartile range) or *n* (%) of cases

*ECPR* extracorporeal cardiopulmonary resuscitation, *NLT* no and low-flow time, *OR* odds ratio, *CI* confidence interval, *BCPR* bystander cardiopulmonary resuscitation, *SR* shockable rhythm, *CPC* cerebral performance category

^a^Defined as the interval from witnessed OHCA to start of reperfusion (start of extracorporeal membrane oxygenation for ECPR or return of spontaneous circulation for CCPR)

**Table 5:** Multivariable analysis of favourable neurological outcome in all CCPR cases and in patients divided by NLT

Variables	All cases (*n* = 2278)	*P* value	OR (95% CI)	NLT > 30 min (*n* = 1362)	*P* value	OR (95% CI)	NLT > 45 min (*n* = 719)	*P* value	OR (95% CI)
Age (years)	77 (66–85)	< 0.001	0.944 (0.932–0.956)	78 (68–85)	0.006	0.958 (0.929–0.988)	78 (78–85)	0.017	0.934 (0.884–0.988)
Male (%)	1457 (64%)	0.553	1.121 (0.768–1.637)	848 (62%)	0.578	0.758 (0.285–2.017)	467 (65%)	0.662	0.660 (0.103–4.245)
BCPR (%)	991 (44%)	< 0.001	3.421 (2.396–4.884)	605 (44%)	0.265	1.695 (0.670–4.290)	313 (44%)	0.246	0.256 (0.025–2.564)
SR (%)	463 (20%)	< 0.001	4.425 (3.037–6.447)	213 (16%)	< 0.001	11.133 (4.122–30.067)	102 (14%)	0.011	9.996 (1.684–59.322)
NLT (min)^a^	36 (21–50)	< 0.001	0.858 (0.843–0.872)	47 (38–57)	0.029	0.951 (0.910–0.995)	56 (50–63)	0.274	0.940 (0.841–1.050)
CPC 1–2	390 (17%)	–	–	20 (2%)	–	–	6 (1%)	–	–

Values are median (interquartile range) or *n* (%) of cases

*CCPR* conventional cardiopulmonary resuscitation, *NLT* no and low-flow time, *OR* odds ratio, *CI* confidence interval, *BCPR* bystander cardiopulmonary resuscitation, *SR* shockable rhythm, *CPC* cerebral performance category

^a^Defined as the interval from witnessed OHCA to start of reperfusion (start of extracorporeal membrane oxygenation for ECPR or return of spontaneous circulation for CCPR)

## Discussion

In the present study, propensity score analysis revealed a negative effect of ECPR on improving the neurological outcomes after OHCA in all eligible cases. However, in patients with NLT of > 30 min and 45 min, showed positive results for favourable outcome (CPC 1–2) (OR 2.977 and 5.099, respectively). Actually, in patients with NLT of ≤ 30 min, showed negative result (OR 0.174). Patients with NLT of ≤ 30 min were 4% (11/307) in ECPR and 40% (916/2278) in CCPR groups, and CCPR with NLT of ≤ 30 min was 35% (916/2585) in all eligible patients. In addition, there were 189 of motor score 6 (Glasgow coma scale) cases after the admitted hospital in CCPR group, 97% (183/189) of them were cases with NLT of ≤ 30 min. These results imply that ECPR could not show the advantage with NLT of ≤ 30 min by propensity score analysis, might be affected by population power and different neurological backgrounds as above, despite the rates of actual favourable outcome (CPC 1–2) were not quite different (ECPR: 44% vs. CCPR: 40%). Anyhow, from the present data, ECPR could not show the effectiveness in the cases, contained CCPR with quick ROSC. ECPR also could not alter the outcome of cases with quick ROSC, which would not be thought as ECPR target.

On the other hand, in subgroup patients with NLT of > 30 min and 45 min, ECPR showed positive results with propensity score analysis, which meant that in cases of prolonged NLT (> 30 min and 45 min), ECPR could show the effectiveness. Therefore, we think that the positive effect of ECPR is due to extension of the therapeutic time window. To investigate this further, we performed multivariable analyses in all cases and in cases with an NLT of > 30 min or > 45 min for the ECPR and CCPR groups separately. Amongst ECPR cases, we found that the NLT was significantly associated with favourable neurological outcomes in all patients and in both subgroups of patients (OR of < 0.900). This is especially notable in the NLT > 45 min subgroup. By comparison, in CCPR cases, we found that the NLT was significant in all patients (OR of 0.858) and in patients with an NLT of > 30 min (OR of 0.951). These results imply that ECPR may be superior to CCPR for patients with an NLT of > 30 min.

Comparing the treatment outcomes between ECPR and CCPR is an important research topic. Because of the difficulty of performing randomised controlled studies and allocating patients to ECPR and CCPR, most insight has come from observational studies. A meta-analysis of observational studies showed the superiority of ECPR over CCPR in terms of the 30-day neurological outcomes and survival [17]. The hospital’s capability in performing ECPR was also associated with favourable neurological outcomes at 1 month [18]. Therefore, ECPR may provide better neurological outcomes compared with CCPR, but the specific effects of ECPR was unclear because other factors, including age, BCPR, shockable rhythm and NLT, are important factors, even in CCPR, and may confound the observed results.

The SAVE-J study showed that ECPR after 15 min of CCPR started within an NLT of 45 min was associated with better neurological outcomes at 6 months in cases of witnessed OHCA with a shockable rhythm [19]. This suggests that the better outcomes of ECPR may be due to extending the therapeutic time window. Interestingly, in a study that compared ECPR and CCPR by propensity score matching [20], there was no difference in the neurological outcomes of ECPR and CCPR (*P* = 0.11). However, because NLT was also used to generate the propensity scores, patient matching did not reveal an advantage of ECPR in prolonging the therapeutic time window (this study used propensity score analysis with matching (1:1) method, which has possibility of making biassed small groups; therefore, we used propensity score analysis with IPTW method in the present study). In another article, the authors compared patients who underwent ECPR in an observational study and patients who underwent standard CCPR with amiodarone in a double-blind randomised controlled study (Amiodarone, Lidocaine, or Placebo study) of witnessed OHCA with a shockable rhythm [21]. The analyses revealed that ECPR was associated with better neurological outcomes compared with CCPR (favourable neurological outcome on discharge: 39% vs 23%) despite longer CPR duration (60 min vs 35 min) [21]. Therefore, the authors concluded that ECPR was effective despite severe progressive metabolic derangement.

Although it is possible that ECPR may prolong the therapeutic time window, shortening the NLT is expected to achieve greater improvements in the neurological outcomes after OHCA. Several reports have indicated that the interval from arrest to ECPR is associated with the outcome of OHCA, and that prehospital cannulation, as part of aggressive ECPR interventions, could improve the neurological outcomes [17, 22].

In our study, we demonstrated the efficacy of ECPR in patients with NLT > 30 min. In patients containing NLT ≤ 30 min, CCPR showed more advantage compare to ECPR, although it is rare in ECPR with NLT ≤ 30 min. ECPR is often performed in combination with TTM. However, as described in the Introduction, there is limited evidence for the efficacy of this combination. In the present study, we excluded any cases who received TTM. Therefore, in the future, it may be necessary to investigate the efficacy of ECPR in combination with TTM.

This study has several limitations. First, although the registry includes a nationwide cohort, the study was performed retrospectively, which may introduce some bias. Second, the neurological outcomes were assessed in terms of CPC at 1 month after resuscitation. It is possible that the neurological outcomes might have changed after 6 months or 1 year. Third, although propensity score analysis demonstrated the efficacy of ECPR, which was shown only in subgroups. Forth, whilst the propensity score analysis demonstrated the efficacy of ECPR in subgroups, other factors might confound the results and introduce some bias. Fifth, cases with TTM were excluded in the present study; however, the reasons of TTM maladaptation were unclear, this could be case selection bias.

## Conclusions

Results of this nationwide Japanese cohort study revealed ECPR was inferior to CCPR in cases containing NLT ≤ 30 min; however, a significant difference was shown in the neurological outcomes between ECPR and CCPR in cases with an NLT of > 30 min, without TTM, after adjusting for NLT.

## Data Availability

The datasets are only available to the study group.

## Abbreviation

ECMO: Extracorporeal membrane oxygenation
ECPR: Extracorporeal cardiopulmonary resuscitation
OHCA: Out-of-hospital cardiac arrest
CCPR: Conventional cardiopulmonary resuscitation
VF: Ventricular fibrillation
VT: Ventricular tachycardia
NLT: No and low flow time
ROSC: Return of spontaneous circulation
TTM: Target temperature management
CPC: The Glasgow–Pittsburgh cerebral performance category
